# Effects of Nordic walking in Alzheimer’s disease: A single-blind randomized controlled clinical trial

**DOI:** 10.1016/j.heliyon.2023.e15865

**Published:** 2023-04-28

**Authors:** A. Angiolillo, D. Leccese, S. Ciccotelli, G. Di Cesare, K. D'Elia, N. Aurisano, C. Matrone, C. Dentizzi, A. Di Costanzo

**Affiliations:** a Centre for Research and Training in Medicine for Aging, Department of Medicine and Health Sciences “Vincenzo Tiberio”, University of Molise, 86100, Campobasso, Italy; b Centre for Cognitive Disorders and Dementias-ASREM, 86100, Campobasso, Italy; c Division of Pharmacology, Department of Neuroscience, School of Medicine, University of Naples Federico II, 80131, Naples, Italy

**Keywords:** Alzheimer’s disease, Nordic walking, Cognitive impairment, Physical activity, Cognitive domains

## Abstract

Non-pharmacological approaches, including exercise programs, have been proposed to improve cognitive function and behavioral symptoms, such as depression, agitation, or aggression, in the management of patients with Alzheimer's disease (AD). Indeed, physical inactivity is one of the main modifiable risk factors in patients with AD, as well as in the development of cardiovascular diseases and related pathologies.

Although Nordic Walking (NW), a particular type of aerobic exercise, is known to benefit the health of aging populations, there is little evidence that patients with AD may benefit from this non-pharmacological treatment. In this context, we performed a pilot study in 30 patients with mild/moderate AD to evaluate whether NW influences different cognitive domains, including executive functions, visual-spatial abilities, and verbal episodic memory. To this aim, 15 patients (Control group, CG) underwent reality orientation therapy, music therapy, motor, proprioceptive and postural rehabilitation, and 15 patients (experimental group, EG) in addition to the activities performed by the CG also had the NW with a frequency of twice a week. Neuropsychological assessments and evaluations of daily activities and quality of life were performed at baseline and after 24 weeks. Twenty-two patients, including 13 in the CG and nine in the EG completed the activity program after 24 weeks. The EG showed a significant improvement in the Frontal Assessment Battery, Rey's auditory Verbal Learning Test Delayed Recall, Raven's Colored Progressive Matrices, and completion time for the Stroop Word-Color Interference test, compared to the CG. NW was able to improve cognitive domains like visual-spatial reasoning abilities, verbal episodic memory, selective attention, and processing speed in AD patients. These results, if confirmed by further studies with a larger number of patients and a longer training period, may prospect NW as a safe and likely useful strategy to slow down cognitive impairment in mild/moderate AD.

## 1 Introduction

Alzheimer's disease (AD) is the most prevalent type of dementia whose main neuropathological features are cerebral amyloidosis and tauopathy. Non-modifiable risk factors include age, APOE-ε4 allele, and family history; modifiable risk factors are essentially cardiovascular risk factors, such as midlife hypertension, midlife obesity, diabetes, smoking, and physical inactivity [1,2]. In the absence of effective disease-modifying therapies for AD, much attention has been paid to the modifiable risk factors, whose recognition upholds primary prevention in the management of dementia. Indeed, the Lancet International Commission on Dementia Prevention, Intervention and Care estimated that interventions on modifiable risk factors could prevent approximately 35% of dementia cases [3].

Longitudinal studies have shown an association between physical activity and the risk of cognitive decline, suggesting that exercise can delay the onset of AD in a dose-dependent manner [1,4]. Furthermore, it is worth noting that also late-onset exercise interventions improve brain health and that physical activity can be beneficial in both brain aging and AD-neurodegeneration [[5], [6], [7]]. Specifically, it has been shown that exercise can increase cerebral blood flow, improve neurotransmitter release thereby facilitating neurogenesis and synaptogenesis, and promote the synthesis of antioxidant molecules, effects that oppose brain changes related to aging and AD [8,9]. Therefore, physical activity has shown promise in the prevention and treatment of AD [10,11].

Nordic walking (NW) is a particular type of aerobic exercise where simple walking is enriched by the use of specifically ergonomically designed poles, with the advantage of active involvement in the upper body and arms [12], resulting in a higher heart rate, oxygen consumption, and caloric expenditure compared to regular walking, and a decrease in the perceived level of exertion [13]. The use of NW poles is valuable, as they provide stability that can promote physical activity, even among the elderly. The effects of NW have been evaluated in several medical conditions such as Parkinson's disease (PD), which seems to play an important role in rehabilitation because of its potential to improve functional mobility, gait, and quality of life [14]. Moreover, recent studies have shown that regular NW training leads to improved cognitive function in older adults [15,16]. To date, a single 3-month NW training study has been conducted in patients with AD, and only a few cognitive domains have been investigated [17]. Therefore, detailed scientific data on the role of NW in AD are still lacking.

Our study aimed to evaluate the effects of 24-week NW training on cognitive function in patients with mild/moderate AD to implement therapeutic strategies for the management of dementia.

## 2 Materials and methods

### 2.1 Study design and patients

The study was a single-blind, randomized, controlled clinical trial involving 30 patients with mild/moderate AD. Patients were enrolled from among individuals accessing the Day Center for Alzheimer's Patients of the Molise Region Health Authority (ASReM) and the Centre for Research and Training in Medicine of Aging (CeRMI) of the University of Molise (Campobasso, Italy). Eligibility criteria were: diagnosis of “probable AD with documented decline”, according to criteria of the NIA-AA (National Institute on Aging and Alzheimer's Association) [18], Mini-Mental State Examination (MMSE) [19] between 18 and 24 for mild AD, MMSE between 9 and 17 for moderate AD, and Clinical Dementia Rating (CDR) scale >1. The exclusion criteria were as follows: Geriatric Depression Scale (GDS) [20] >6 and inability to perform a 6-min walk test [21], as well as the presence of other comorbidities that prevented adherence to the physical activity program. All the patients underwent blood tests and brain imaging to rule out other possible causes of dementia. Patients with multiple or extensive infarcts or severe white matter hyperintensities were excluded, as were those with other pathological findings such as meningioma, glioma, subdural hematoma, vascular malformation, or normal pressure hydrocephalus.

The study was conducted in accordance with the ethical principles of the Declaration of Helsinki and approved national and international guidelines for human research. The Ethics Committee of ASReM reviewed and approved this study (protocol n. 142–19/09/2019). Written informed consent was obtained from all the participants or their caregivers.

### 2.2 Randomization and activity program

The patients were randomly divided into two groups of 15 patients according to a computer-generated randomization list: the control group (CG) and the experimental group (EG). Both CG and EG patients underwent 2 h a week of cognitive re-education based on formal Reality Orientation Therapy (ROT) with physiotherapists or occupational therapists; 8 h a week of cognitive re-education based on informal ROT with psychologists and socio-health professionals; 4 h a week of music therapy with music therapists; 2 h a week of motor, proprioceptive and postural rehabilitation with physiotherapists. The 15 EG patients performed training sessions of 60 min twice a week, under the supervision of a specialized trainer, consisting of 10 min of warm-up with stretching exercises, 40 min of NW, and 10 min of cool down with stretching exercises. NW training was not standardized in terms of intensity level but was carried out on the personal perception of maximum exertion. All activities were performed for 24 weeks.

### 2.3 Neuropsychological assessment and evaluation of daily activities and quality of life

All patients underwent extensive neuropsychological assessment (Table 1), including the MMSE, Frontal Assessment Battery (FAB) [22], immediate and delayed recalls in the Rey's Auditory Verbal Learning Test (RAVLT) [23], Prose Memory Test (PMT) [24], Attentional Matrices Test [24], Raven's Colored Progressive Matrices (CPM) [25], completion time and errors in the short version of the Stroop Word-Color Interference test (SWCT) [26,27], and Copying Geometric Drawings (CGD) [24]. All raw test scores were adjusted for age, educational level, and sex. Activities of daily living (ADL), instrumental activities of daily living (IADL), and quality of life in Alzheimer's disease (QoL-AD) [28] were also assessed. The 24-week change in the MMSE score was the primary outcome, whereas the 24-week change in the remaining assessment scores was the secondary outcome.

**Table 1:** Neuropsychological assessment used in the study.

TEST	Description	Score
MMSE	Mini Mental State Examination: global cognitive assessment	Score 0–30; 0-8: severe cognitive impairment 9-17: moderate cognitive impairment 18-24: mild cognitive impairment
FAB	Frontal Assessment Battery: executive functions assessment	Score 0–18; pathological score ≤12.03
RVLT-I	Rey's auditory Verbal Learning Test-Immediate Recall: verbal episodic memory (immediate recall)	Score 0–75; pathological score ≤28.53
RVTL-D	Rey's auditory Verbal Learning Test-Delayed Recall: verbal episodic memory (delayed recall)	Score 0–15; pathological score ≤4.69
CPM	Raven's Colored Progressive Matrices: visual-spatial reasoning abilities	Score 0–36; pathological score ≤18.96
SWCT-time	Stroop Word-Color Interference test: selective attention and processing speed assessment	Pathological score ≥36.92
SWCT-errors	Stroop Word-Color Interference test: cognitive flexibility and ability to inhibit cognitive interference	Pathological score ≥4.24
Attentional Matrices	Visual attention assessment	Score 0–60; Pathological score ≤30
PMT	Prose Memory test: verbal memory assessment	Score 0–28; Pathological score ≤7.5
CGD	Copying Geometric Drawings: visual spatial ability	Pathological score ≤7.17
QoL-AD	Quality of life in Alzheimer's Disease: quality of life assessment in adults with cognitive impairment	Score: 0–100; the higher the score, the better the perceived quality of life
ADL	Activity of Daily Living: independence in activities of daily living	Score 0–6: the higher the score, the lower the independence of patient
IADL	Instrumental Activity of Daily Living independence in instrumental activities of daily living	Score 0–8: the higher the score, the lower the independence of patient

### 2.4 Statistical analysis

Data were analyzed using the SPSS Statistics software (version 17.0; SPSS Inc., Chicago, IL, USA). Variables were examined using box and normal quantile-quantile plots, Shapiro-Wilk and Kolmogorov-Smirnov tests, and when a normal distribution could not be accepted, variable transformations (square, square root, logarithmic, reciprocal of square root or reciprocal transformations) were analyzed. Normally distributed continuous variables were reported as arithmetic means and SDs and analyzed using the independent-samples *t*-test, while non-normally distributed continuous variables were reported as median and interquartile range and analyzed using the non-parametric Mann-Whitney *U* test. Categorical variables were examined using the chi-square test (χ2). Differences between baseline and 24-week assessment scores (24-week changes) were calculated in each group (CG and EG) and compared using the Mann-Whitney *U* test. Statistical significance was set at P < 0.05.

## 3 Results

**Fig. 1:**
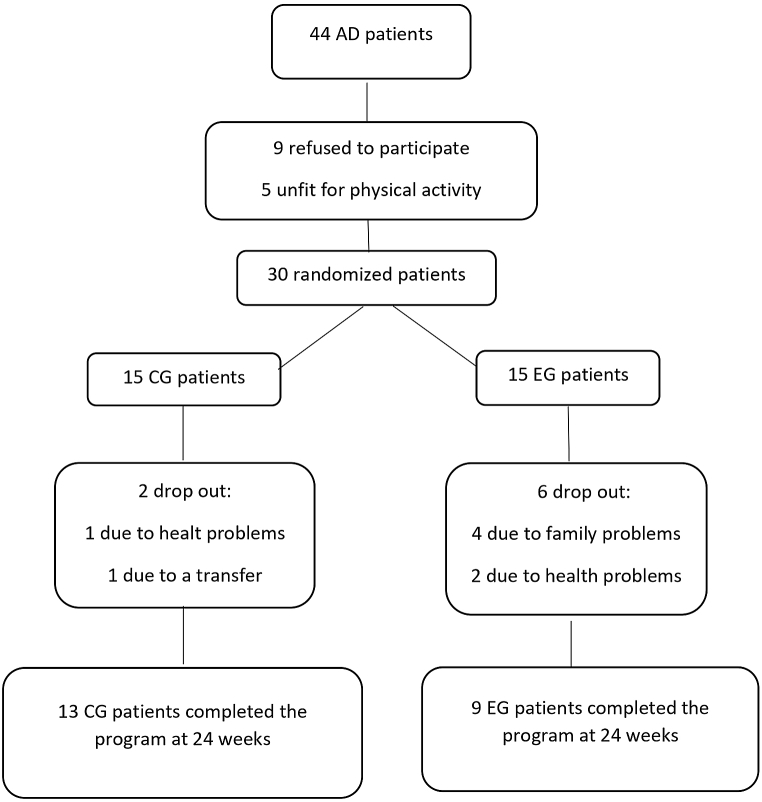
Flow diagram of the study.

Twenty-two of the 30 patients, nine in the EG and 13 in the CG, completed the activity program at 24 weeks and were included in the final analysis (Fig. 1). There were no significant differences between the two groups in terms of clinical or demographic characteristics (Table 2), as well as in term of baseline assessment scores (Table 3).

**Table 2:** Demographic and clinical features of patients with AD.

	CG (N. 13)	EG (N. 9)	p
Females/males (N.)	8/5	6/3	0.82
Age (mean ± SD, y)	78.92 + 8.04	78.89 + 6.68	0.99
Education level (mean ± SD, y)	8.31 + 5.17	7.89 + 5.49	0.86
BMI (mean ± SD, kg/m^2^)	25.20 + 2.16	27.00 + 2.66	0.09
Medical History (N; %)			
Smoke	2; 15.3%	3; 33.3%	0.30
Dyslipidemia	2; 15.3%	2; 22.2%	0.68
Diabetes	1; 7.7%	2; 22.2%	0.32
Hypertension	6; 46.1%	2; 22.2%	0.25
Myocardial infarction	0	1; 11.1%	0.21
TIA/Stroke	0	0	
Drugs (N; %)			
Antihypertensive	4; 30.7%	2; 22.2%	0.89
Lipid-lowering	2; 15.3%	2; 22.2%	0.46
Hypoglycemic	1; 7.7%	2; 22.2%	0.32
Memantine	7; 53.8%	3; 33.3%	0.18
AChE-I	7; 53.8%	4; 44.4%	0.20

CG: control group; EG: experimental group; BMI: body mass index; TIA: transient ischemic attack; AChE-I: acetylcholinesterase inhibitors.

**Table 3:** Assessment scores at baseline and 24 weeks, and the extent of changes in all patients completing the 24 week-program activity.

	CG (13 patients)			EG (9 patients)			
	Baseline	24 weeks	Changes	Baseline	24 weeks	Changes	p
MMSE	17.49 (±3.89)^a^	15.31 (±5.79)^a^	1.00 (±3.00)	20.24 (±1.99)^a^	20.37 (±3.60)^a^	−0.82 (±4.63)	0.20
FAB	14.31 (±3.81)^a^	10.34 (±5.02)^a^	3.00 (±2.85)	10.88 (±4.63)^a^	12.99 (±3.46)^a^	−2.00 (±5.40)	0.04*
RVLT-I	16.48 (±5.96)^a^	17.12 (±7.72)^a^	−4.00 (±5.90)	21.13 (±4.18)^a^	23.66 (±5.74)^a^	−4.00 (±3.35)	0.77
RVLT-D	3.96 (±1.33)^a^	1.77 (±2.18)^a^	3.10 (±3.40)	2.88 (±1.08)^a^	2.99 (±1.09)^a^	0.00 (±0.00)	0.02*
CPM	20.22 (±4.37)^a^	18.47 (±6.61)^a^	0.50 (±1.00)	20.33 (±4.01)^a^	24.93 (±6.23)^a^	−3.35 (±2.75)	0.002*
SWCT-time	33.28 (±24.18)^a^	81.99 (±30.05)^a^	−39.75 (±17.99)	59.13 (±65.43)^a^	31.04 (±36.27)^a^	57.78 (±67.92)	0.006*
SWCT-errors	8.13 (±8.06)^b^	17.50 (±6.75)^b^	−4.88 (±5.31)	7.50 (±18.75)^b^	4.75 (±8.00)^b^	0.38 (±8.00)	0.11
Attentional Matrices	33.59 (±6.59)^a^	26.70 (±11.54)^a^	5.00 (±9.63)	28.69 (±10.84)^a^	27.08 (±8.50)^a^	−0.50 (±12.50)	0.30
PMT	0.00 (±3.00)^b^	0.00 (±1.25)^b^	0.00 (±0.05)	0.35 (±3.75)^b^	1.50 (±0.75)^b^	0.75 (±3.00)	0.88
CGD	8.16 (±4.04)^a^	7.42 (±4.69)^a^	0.50 (±1.00)	8.72 (±3.35)^a^	8.83 (±4.62)^a^	1.00 (±1.75)	0.62
QoL-AD	28.15 (±8.82)^a^	28.38 (±9.36)^a^	0.00 (±4.0)	31.14 (±7.46)^a^	26.16 (±9.35)^a^	6.00 (±14.69)	0.30
ADL	2.00 (±3.00)^b^	2.00 (±3.00)^b^	0.00 (±0)	5.00 (±3.00)^b^	5.00 (±3.00)^b^	0.00 (±1.00)	0.35
IADL	1.00 (±2.00)^b^	1.00 (±2.00)^b^	0.00 (±0)	2.00 (±4.00)^b^	1.00 (±1.00)^b^	0.00 (±1.00)	0.19

Values are reported as mean ± standard deviation (^a^), or median ± interquartile range (^b^). Changes: differences between baseline and 24-week assessment scores. P, significance of Mann-Whitney *U* test comparing the 24-week changes. Baseline assessment scores did not show significant differences between groups (p ≥ 0.07). *p-value <0.05. EG: experimental group; CG: control group; MMSE: Mini Mental State Examination; FAB: Frontal Assessment Battery; RVLT-I: Rey's auditory Verbal Learning Test - Immediate Recall; RVTL-D: Rey's auditory Verbal Learning Test - Delayed Recall; CPM: Raven's Colored Progressive Matrices; SWCT: Stroop Word-Color Interference test; PMT: Prose Memory test; CGD: Copying Geometric Drawings; QoL-AD: Quality of life in Alzheimer's Disease; ADL: Activity of Daily Living; IADL: Instrumental Activity of Daily Living.

**Fig. 2:**
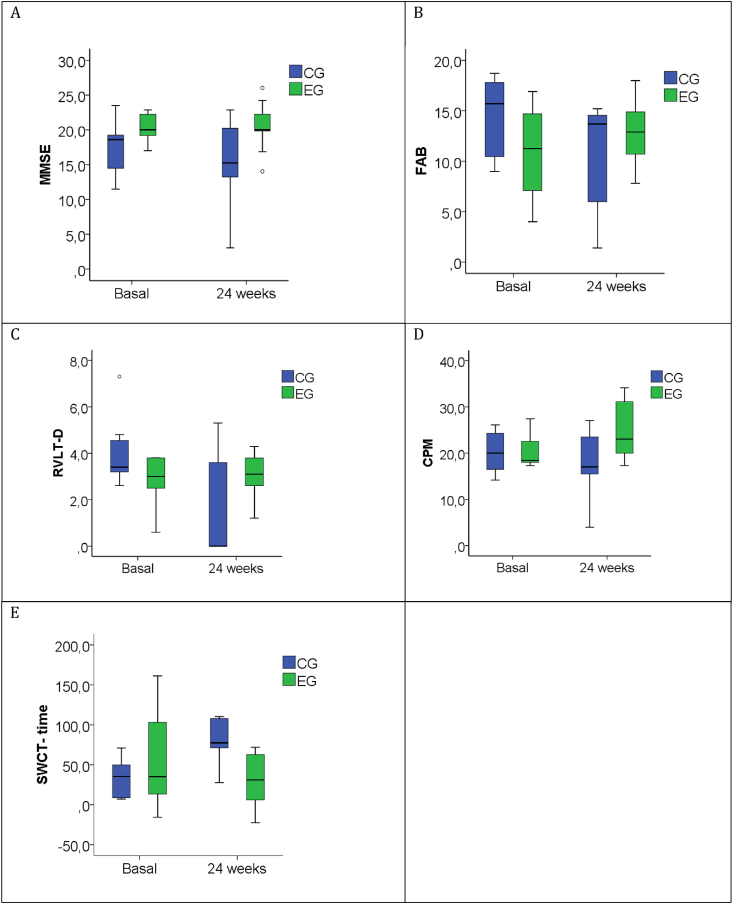
Neuropsychological test scores at baseline and 24 weeks in all patients completing the program activity. Box-plots show median (horizontal line in the box), 25th and 75th percentiles (edges of box), maximum and minimum values (whiskers) and outliers (°). Mini Mental State examination scores appeared substantially unchanged in the experimental group (EG), while tended to decrease in the control group (CG), but the 24-week changes did not show significant differences (A). The EG showed an improvement in Frontal Assessment Battery (FAB) (B), Rey's auditory Verbal Learning Test Delayed Recall (RVTL-D) (C), Raven's Colored Progressive Matrices (CPM) (D) and in completion time for Stroop Word-Color Interference test (SWCT) (E), compared to the CG.

The results of the baseline and 24-week assessments and related 24-week changes are shown in Table 3. The 24-week changes in MMSE scores did not show significant differences between the groups (Table 3), even though scores tended to decrease in the CG, whereas they appeared substantially unchanged in the EG (Table 3; Fig. 2A). The 24-week changes in FAB, RAVLT delayed recall, SWCT completion time, and CPM scores showed a significant improvement in the EG, while the remaining assessment scores (RAVLT immediate recall, Attentional Matrices test, PMT, CGD, SWCT errors, QoL-AD, ADL, and IADL) did not show significant differences between the groups (Table 3). In particular, the RVLT delayed recall scores decreased in the CG but remained substantially unchanged in the EG (Table 3; Fig. 2C), while FAB (Table 3; Fig. 2B), CPM (Table 3; Fig. 2D), and completion time for the SWCT (Table 3; Fig. 2E) showed a worsening in the CG and an improvement in the EG. Since in the EG only patients with mild AD (MMSE between 22.86 and 17.03) were present, we repeated the statistical analysis comparing the patients with mild AD in both groups: 9 patients in the EG and 8 patients in the CG (MMSE between 23.49 and 17.03). The results are reported in Table 4 and were similar to those reported in Table 3, with the exception of the FAB scores, which did not show significant differences between the groups.

**Table 4:** Assessment scores at baseline and 24 weeks, and the extent of changes in patients with mild AD after 24-week program activity.

CG (8 patients)				EG (9 patients)			
	Baseline	24 weeks	Changes	Baseline	24 weeks	Changes	p
MMSE	20.09 (±2.35)^a^	17.30 (±6.44)^a^	2.79 (±5.61)	20.24 (±1.99)^a^	20.37 (±3.60)^a^	−0.82 (±4.63)	0.245
FAB	14.7 (±3.38)^a^	12.56 (±3.28)^a^	2.14 (±5.08)	10.88 (±4.63)^a^	12.99 (±3.46)^a^	−2.00 (±5.40)	0.202
RVLT-I	16.89 (±6.26)^a^	20.14 (±6.22)^a^	−3.26 (±6.51)	21.13 (±4.18)^a^	23.66 (±5.74)^a^	−4.00 (±3.35)	0.447
RVLT-D	4.37 (±1.50)^a^	2.31 (±2.37)^a^	2.06 (±1.98)	2.88 (±1.08)^a^	2.99 (±1.09)^a^	0.00 (±0.00)	0.017*
CPM	21.59 (±4.01)^a^	21.73 (±4.59)^a^	−014 (±0.90)	20.33 (±4.01)^a^	24.93 (±6.23)^a^	−3.35 (±2.75)	0.006*
SWCT-time	36.75 (±29.38)^a^	82.73 (±32.85)^a^	−40.53 (±18.84)	59.13 (±65.43)^a^	31.04 (±36.27)^a^	57.78 (±67.92)	0.011*
SWCT-errors	7.00 (±5.75)^b^	17.50 (±7.63)^b^	−3.25 (±4.25)	7.50 (±18.75)^b^	4.75 (±8.00)^b^	0.38 (±8.00)	0.201
Attentional Matrices	35.504 (±5.59)^a^	31.25 (±6.27)^a^	3.79 (±5.67)	28.69 (±10.84)^a^	27.08 (±8.50)^a^	−0.50 (±12.50)	0.523
PMT	0.0 (±2.68)^b^	0.25 (±2.63)^b^	0.00 (±0.10)	0.35 (±3.75)^b^	1.50 (±0.75)^b^	0.75 (±3.00)	0.669
CGD	9.68 (±3.99)^a^	9.00 (±4.62)^a^	−0.39 (±1.47)	8.72 (±3.35)^a^	8.83 (±4.62)^a^	1.00 (±1.75)	0.281
QoL-AD	28.50 (±9.44)^a^	29.21 (±10.22)^a^	−0.71 (±5.76)	31.14 (±7.46)^a^	26.16 (±9.35)^a^	6.00 (±14.69)	0.211
ADL	2.00 (±3.00)^b^	2.00 (±3.00)^b^	0.00 (±0)	5.00 (±3.00)^b^	5.00 (±3.00)^b^	0.00 (±1.00)	0.633
IADL	1.00 (±3.25)^b^	1.00 (±3.25)^b^	0.00 (±0)	2.00 (±4.00)^b^	1.00 (±1.00)^b^	0.00 (±1.00)	0.300

Values are reported as mean ± standard deviation (^a^), or median ± interquartile range (^b^). Changes: differences between baseline and 24-week assessment scores. P, significance of Mann-Whitney *U* test comparing the 24-week changes. Baseline assessment scores did not show significant differences between groups (p ≥ 0.07). * p-value <0.05. EG: experimental group; CG: control group; MMSE: Mini Mental State Examination; FAB: Frontal Assessment Battery; RVLT-I: Rey's auditory Verbal Learning Test - Immediate Recall; RVTL-D: Rey's auditory Verbal Learning Test - Delayed Recall; CPM: Raven's Colored Progressive Matrices; SWCT: Stroop Word-Color Interference test; PMT: Prose Memory test; CGD: Copying Geometric Drawings; QoL-AD: Quality of life in Alzheimer's Disease; ADL: Activity of Daily Living; IADL: Instrumental Activity of Daily Living.

## 4 Discussion

The results of the present study suggest that NW can improve cognitive function in patients with AD. To the best of our knowledge, this is the first study to investigate the role of a 24-week NW training program on cognitive function in patients with AD, using an extensive neuropsychological battery. NW was able to enhance the executive functions investigated by FAB, selective attention and processing speed investigated by SWCT, visual-spatial reasoning abilities investigated by CPM, and verbal episodic memory investigated by RTVL. However, by restricting the analysis to only patients with mild AD, significant improvement in FAB disappeared. These results are consistent with previous studies showing that NW training improved executive function, spatial memory, and information processing speed response time in older adults [16], as well as processing speed and selective attention in elderly women [15]. MMSE scores appeared substantially unchanged in the EG but tended to decrease in the CG, but the 24-week MMSE changes, the primary outcome of the present study, did not show significant differences between groups (Fig. 2; Table 2). This result is in line with a recent study that investigated the effects of 3-month NW training on the Montreal Cognitive Assessment scale (MoCA), another tool for the assessment of global cognitive function, in patients with AD [17]. Contrary to other studies that have shown an improvement in the quality of life in the elderly following NW training [29,30], we found no differences in QoL-AD between the CG and EG. It should be emphasized that it is not always simple to establish the quality of life in patients with AD, as anosognosia often coexists, or caregivers may have an altered perception of their quality of life. In line with Górniak et al. [17], no significant improvement was observed in ADL and IADL scores (Table 2).

The main limitations of our study are the small sample size and the lack of standardization of NW intensity. Further studies conducted on a larger sample and with a longer training period are needed to clarify if the positive effects of NW on the cognitive functioning of AD patients continue to exist, given the slowly progressive course of the disease.

Several mechanisms have been proposed to explain the enhanced cognitive performance in response to physical activity. Shimada et al. [31] evaluated the effects of exercise on brain activity in healthy older adults and found that walking increased regional glucose metabolism in specific brain areas, such as the left posterior entorhinal and precuneus cortices, which play important roles in episodic and visuospatial memory. Erickson et al. [32] demonstrated that physical activity was associated with an increase in grey matter volume in the same regions involved in age-related atrophy, including the prefrontal cortex and hippocampus. Voelcker-Rehage et al. (2011) showed that cardiovascular and coordination exercises improve cognitive function in older adults in different ways. Specifically, cardiovascular training can activate the sensorimotor cortex, whereas coordination training improves the visuospatial network [33]. As a matter of fact, NW can be considered a multicomponent exercise including both cardiovascular activity and motor coordination between the upper and lower limbs [34].

To date, there is growing evidence that vascular endothelial growth factor (VEGF) and brain-derived neurotrophic factor (BDNF) are the major factors behind the effects of physical activity on the brain [35]. VEGF is a potent vasogenic and angiogenic factor, whose signaling pathway alterations occur in the AD neuropathological cascade [36]. Izzicupo et al. analyzed VEGF levels in postmenopausal women after NW training and showed that NW increased circulating VEGF levels more than regular walking at the same training intensity [37]. BDNF is a potent neurotrophin involved in synaptic plasticity, learning, and memory, and it is well established that acute physical activity increases its circulating levels [38]. However, Domaszewska et al. found no differences in BDNF levels in postmenopausal women undergoing NW [39], whereas Walentukiewicz et al. showed a decrease in circulating BDNF levels in elderly women after vitamin D supplementation and NW training [40].

Several studies have shown that NW training also induces positive changes in the lipid profile of elderly women supplemented with vitamin D [41], and in overweight and obese subjects [42]. Specifically, NW training induces a decrease in total blood cholesterol, low-density lipoprotein cholesterol, and triglyceride levels [41]. Moreover, the 12-week NW training undertaken by premenopausal women had a positive effect on the antioxidant capacity of plasma [43]. These assumptions are noteworthy since lipid profile alterations and oxidative stress are closely linked to AD [44,45].

Recent meta-analyses have shown that physical exercise can slow cognitive decline in older adults with AD [7,46]. However, it should be emphasized that discrepancies exist and the effects of exercise on cognitive functioning in patients with dementia have not always been revealed in clear evidence [47]. Moreover, there is no consistent agreement on the type of intensity, exercise, number of sessions, and duration that could have the best effects on cognitive function in patients with AD.

## 5 Conclusions

The 24-week NW training resulted in improvements in visual-spatial reasoning abilities, verbal episodic memory, selective attention, and processing speed in patients with AD.

Although the study was carried out in a small number of patients and for a relatively short duration, it prospects NW for a potentially safe and effective strategy to decelerate cognitive decline in the mild/moderate stages of AD in combination with other approaches.

Indeed, further studies with a larger sample size of patients and a longer training period will be needed to clarify if the positive effects of NW on cognitive functions of patients with AD may last long, given the slowly progressive course of the disease.

## Author contribution statement

Angiolillo A.: Analyzed and interpreted the data; Wrote the paper.

Leccese D.: Analyzed and interpreted the data.

Ciccotelli S., Di Cesare G., D'Elia K.: Performed the experiments.

Aurisano N.: Conceived and designed the experiments; Performed the experiments.

Matrone C.: Contributed reagents, materials, analysis tools or data; Wrote the paper.

Dentizzi C.: Conceived and designed the experiments.

Di Costanzo A.: Conceived and designed the experiments; Analyzed and interpreted the data; Contributed reagents, materials, analysis tools or data; Wrote the paper.

## Funding statement

This Research was funded by thea Ricerca PRIN 20172017T9JNLT(2017T9JNLT).PRIN 2017 (2017T9JNLT) (Italian Ministry of Education, University and Research) 10.13039/501100003407a Ricerca (2017T9JNLT).

## Data availability statement

Data will be made available on request.

## Declaration of competing interest

The authors declare that they have no known competing financial interests or personal relationships that could have appeared to influence the work reported in this paper.
